# Implementation Frameworks for Artificial Intelligence Translation Into Health Care Practice: Scoping Review

**DOI:** 10.2196/32215

**Published:** 2022-01-27

**Authors:** Fábio Gama, Daniel Tyskbo, Jens Nygren, James Barlow, Julie Reed, Petra Svedberg

**Affiliations:** 1 School of Business, Innovation and Sustainability Halmstad University Halmstad Sweden; 2 School of Administration and Economic Science Santa Catarina State University Florianópolis Brazil; 3 School of Health and Welfare Halmstad University Halmstad Sweden; 4 Centre for Health Economics and Policy Innovation Imperial College Business School London United Kingdom

**Keywords:** implementation framework, artificial intelligence, scoping review

## Abstract

**Background:**

Significant efforts have been made to develop artificial intelligence (AI) solutions for health care improvement. Despite the enthusiasm, health care professionals still struggle to implement AI in their daily practice.

**Objective:**

This paper aims to identify the implementation frameworks used to understand the application of AI in health care practice.

**Methods:**

A scoping review was conducted using the Cochrane, Evidence Based Medicine Reviews, Embase, MEDLINE, and PsycINFO databases to identify publications that reported frameworks, models, and theories concerning AI implementation in health care. This review focused on studies published in English and investigating AI implementation in health care since 2000. A total of 2541 unique publications were retrieved from the databases and screened on titles and abstracts by 2 independent reviewers. Selected articles were thematically analyzed against the Nilsen taxonomy of implementation frameworks, and the Greenhalgh framework for the nonadoption, abandonment, scale-up, spread, and sustainability (NASSS) of health care technologies.

**Results:**

In total, 7 articles met all eligibility criteria for inclusion in the review, and 2 articles included formal frameworks that directly addressed AI implementation, whereas the other articles provided limited descriptions of elements influencing implementation. Collectively, the 7 articles identified elements that aligned with all the NASSS domains, but no single article comprehensively considered the factors known to influence technology implementation. New domains were identified, including dependency on data input and existing processes, shared decision-making, the role of human oversight, and ethics of population impact and inequality, suggesting that existing frameworks do not fully consider the unique needs of AI implementation.

**Conclusions:**

This literature review demonstrates that understanding how to implement AI in health care practice is still in its early stages of development. Our findings suggest that further research is needed to provide the knowledge necessary to develop implementation frameworks to guide the future implementation of AI in clinical practice and highlight the opportunity to draw on existing knowledge from the field of implementation science.

## Introduction

### Background

Artificial intelligence (AI) can potentially transform health care data into meaningful and actionable insights [1]; however, AI has not yet become widespread in health care practice. This gap in translation from research to practice is largely owing to the challenges in the implementation of AI [2,3]. This paper aims to assess the current state of academic knowledge relating to the implementation of AI and to appraise the extent to which this knowledge draws from and contrasts with knowledge about general health care technology implementation.

The potential benefits to patients from new health technologies are often missed owing to slow and variable uptake in practice [4]. The emergent field of implementation science has generated many insights into the barriers to and facilitators of the effective uptake and deployment of new health technologies [5], recognizing the unique challenges of intervening in complex health care systems compared with other industrial settings. Typical outputs from implementation science are generalizable implementation theories, models, and frameworks that are developed to describe the factors influencing implementation, predict the conditions required for successful implementation, and provide guidance for conducting and evaluating implementation efforts in health care [5-7]. These frameworks provide a general understanding of the challenges of introducing novel technologies in health care settings.

Importantly, for the development of AI technologies, implementation science has evidenced that passive approaches to the dissemination and diffusion of health care technologies are rarely effective [8]. Instead, purposive implementation efforts are required to mainstream innovation within an organization or health care system [9]. However, to date, most of the research literature on AI in health care deals with the development, application, and evaluation of advanced analytic techniques and models [10-12], primarily within computer science, engineering, and medical informatics. The literature on the implementation of AI to improve existing clinical workflows is more fragmented and mostly based on nonempirical data from proof-of-concept studies [1,13] across multiple subject areas, such as data governance [14], ethics [15], accountability [3], interpretability [16], and regulation [17]. This means that there are uncertainties around factors that influence the implementation of AI in real-world health care setups [10] and that health care professionals lack guidance on how to implement AI in their daily practices [18].

If the value of AI technologies is to be realized in practice, it is important to develop evidence-based approaches to AI implementation. Although it is likely that generalizable implementation theories, models, and frameworks will be able to provide valuable guidance for the implementation of AI technologies, it is likely that the nature of AI features will add new layers of complexity and pose additional challenges to effective implementation [2]. First, AI differs in its potential to augment or constrain the work of health care professionals compared with other technologies. This difference shifts attention away from predicting successful implementations of *passive* technologies (eg, telehealth or pedant alarms) to understand how health care professionals and AI interact to create value for patients and other users. Second, AI challenges our dichotomic belief that divides the realms of human aptitudes and machine capabilities. Recent AI developments allow the perception of emotions, conversations, and, ultimately, creativity. Such capabilities allow AI to enter into domains that were previously exclusive to humans. Third, AI implementation is highly complex, requiring activities that cover a wide range of stakeholders from technology developers, system regulators, organizations and individuals, professionals, patients, and caregivers. This puts AI at the more complex end of what has been studied by implementation sciences, which tend to be well-defined and bounded interventions. Combined, these differences suggest the need to develop an AI-specific evidence base for implementation, and for generalizable knowledge about AI implementation to be shared in AI-specific implementation theories, models, and frameworks.

### Objectives

This study aims to explore the current state of academic knowledge of AI implementation by assessing any implementation theories, frameworks, or models that are specific to AI translation into health care practice. The study objectives are to assess the following:

1. What, if any, AI-specific implementation frameworks in health care exist?
2. How do these AI-specific implementation frameworks draw on and compare to more generalized implementation frameworks for health technologies?
3. What do any AI-specific implementation frameworks reveal about the challenges of AI implementation?

## Methods

### Study Design

An interpretative scoping review was considered the most appropriate method to answer the research questions, as it provides a systematic synthesis of knowledge within a defined area, and with the aim of exploring and mapping key concepts, available evidence, and shortcomings in existing research [19,20]. The following steps were taken: (1) deciding on definitions, (2) systematically searching in databases, (3) selecting and screening studies, and (4) extracting and analyzing the data.

### Deciding on Definitions

The operational definitions for the term implementation, framework, and AI are listed in Table 1. These broad terms were used for searches to cover any literature, specifically using the terms in combination. Using broad terms for the searches instead of more specialized exemplifying terms, the review aims to cover all literature on implementation frameworks for AI translation into health care practice that is framed within these broad terms and thus could be interpreted as generally applicable to this context. Our operational definition for implementation was inspired by Greenhalgh et al [9], which refers to an “active and planned effort to mainstream an innovation within an organization.” According to this definition, implementation efforts are purposeful and are described in sufficient detail such that independent observers can recognize the presence and strength of active and planned actions. Although other definitions of implementation exist, we chose this definition, as it is broad and enables us to identify multiple elements from prior AI-related studies.

**Table 1:** Operational definitions for key concepts.

Term	Operational definition	Examples in health care
Implementation	An intentional effort designed to change or adapt or uptake interventions into routines [9].	Adoption of heart failure prediction software Change the clinical decision support system
Artificial intelligence	A general purpose technology based on a core set of capabilities and computational algorithms designed to mimic human cognitive functions to analyze complex data [2].	Machine learning for mortality prediction Unstructured image data analysis for radiology
Framework	A simplification structure, overview, system or plan of multiple descriptive categories or elements (ie, constructs, concepts, and variable) that streamline the interpretation of a phenomenon [21].	NASSS^a^ framework (for health and care technologies) [5] SHIFT^b^ evidence [7]

^a^NASSS: nonadoption, abandonment, scale-up, spread, and sustainability.

^b^SHIFT: successful health care improvement from translating evidence.

### Systematically Searching in Databases

**Figure 1:**
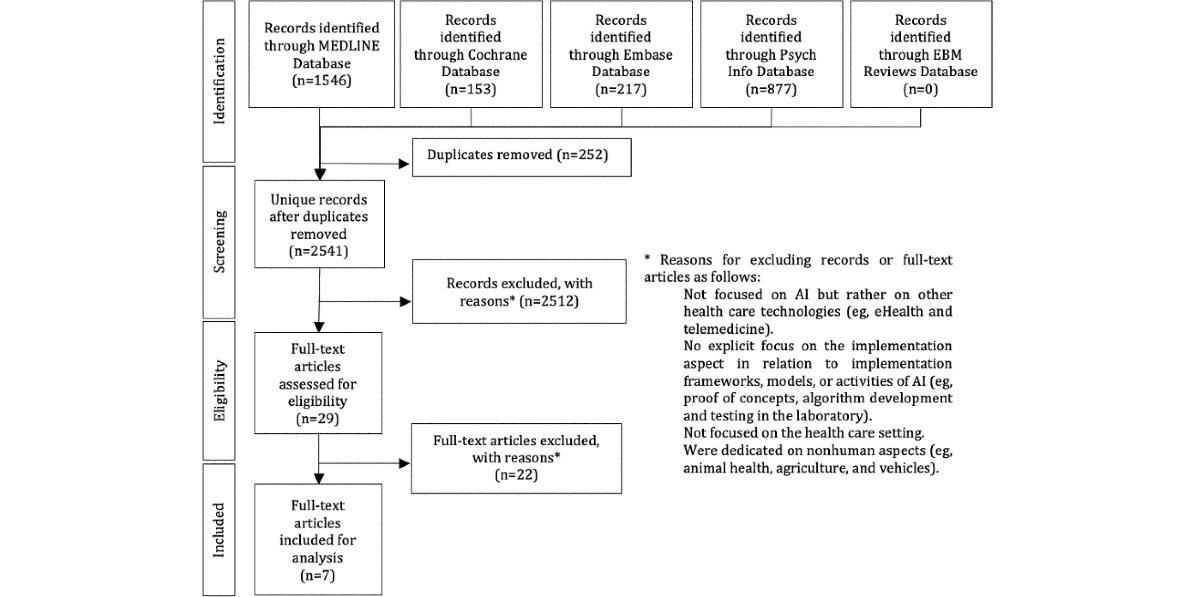
PRISMA (Preferred Reporting Items for Systematic Reviews and Meta-Analyses) flow diagram showing the review process. AI: artificial intelligence; EBM: Evidence Based Medicine.

A systematic search of MEDLINE, Embase, EBM *Reviews,* PsycINFO, and Cochrane databases was performed following the PRISMA (Preferred Reporting Items for Systematic Reviews and Meta-Analyses) flow diagram (Figure 1). The search was limited to the literature published from January 1, 2000, to March 1, 2020, and all original publications that described AI-specific implementation frameworks based on health care studies. Search terms included a combination of terms relating to implementation (implementat*), frameworks (*model* and *theory*), AI (*artificial intelligence*), and machine learning (*ML*; Multimedia Appendix 1). Several different terms could potentially be used as synonymous with the term AI and included as search terms. However, as the purpose of this study was to explore the literature on AI-specific implementation frameworks, our starting point was to include only those studies that presented such frameworks specifically in relation to the overarching concepts of AI and ML. A similar strategy was used by Wolff et al [22] to define the search terms. The MeSH (Medical Subject Headings) vocabulary was used to accurately define the search terms, and the MeSH terms supervised and unsupervised ML were both captured by the term *machine learning*. This strategy was motivated by preliminary literature reviews on AI, consultation with AI experts, and librarians.

### Selecting and Screening Studies

Studies were eligible for inclusion if they were written in English and referred to the implementation of AI (eg, ML) in health care settings. All study designs or publication types were eligible for inclusion to identify the presence of implementation frameworks to guide the use of AI in health care. Conference abstracts, editorials, and technical reports were excluded. Other reasons for exclusion included studies that did not focus on AI, had no explicit focus on implementation in relation to implementation frameworks, models, or theories of AI, that were not focused on the health care setting, or were dedicated to nonhuman aspects (eg, animal health). Titles and abstracts were screened for inclusion by 2 independent reviewers (FG and DT) using the Rayyan web platform. Disagreements were resolved by consensus, and when necessary, a third reviewer was involved (JB). The agreement score during screening was substantial (κ score>0.8).

### Extracting and Analyzing the Data

A 4-step process was used for data extraction and analysis according to the analytical framework of Arksey et al [19]. First, 4 reviewers (FG, DT, JN, and PS) independently piloted a structured data extraction tool on the same final included articles. The reviewers discussed and compared their analyses, and any disagreements were resolved via discussion and consensus. Following the guidelines by Arksey et al [19], information related to country, study design, area of practice, target population, study focus, study aims, and any literature cited as informing the framework development was extracted to understand the main areas of interest.

Second, as this field of research is relatively new, we considered it important to perform a quality assessment of the included articles, even if this is not typical for scoping reviews [19,23]. Thus, we have deliberately addressed concerns that the lack of quality assessment in scoping reviews makes the interpretation and translation of the findings more challenging [24]. The quality of the selected studies was assessed using the Critical Appraisal Skills Programme (CASP) [25]. Two authors (FG and PS) independently rated the articles and resolved disagreements through consensus (Multimedia Appendix 2 [26-32]). The CASP appraisal checklist includes multiple study designs and was therefore chosen as the most appropriate tool to evaluate the selected articles. Articles were not excluded owing to poor quality, as suggested by other studies [33]. Instead, we considered the relative contribution from high and low-quality studies in the analysis phase when discussing the presence of implementation frameworks and elements for AI implementation.

Third, to thematically analyze the literature, the frameworks used in the included studies were categorized in accordance with the Nilsen taxonomy of 5 categories of implementation frameworks [21]. These categories include process models, determinant frameworks, classic theories, implementation theories, and evaluation frameworks. This conceptual foundation is important, as it helps to understand the challenges associated with implementation. Process models aim to guide or describe the process of translating research into practices (eg, stages and phases). Determinant frameworks intend to explain or understand the influence of variables on implementation outcomes (eg, barriers and enablers). Determinant frameworks account for 5 types of determinants that focus on the characteristics of individual elements, including objects (eg, AI), users or adopters, end user (eg, patients), context, and strategy. Both classic theories and implementation theories are distinguished from research-to-practice models, although they explain how change occurs without ambitions to bring about the change. The differences between these 2 theories are that classic theories have been developed from a field external to implementation science (ie, psychology and sociology) and implementation theories have been developed by researchers in the field of implementation science. What all theories have in common is that they have attempted to have some predictive capacity and explain causal mechanisms. Evaluation frameworks specify aspects of implementation to evaluate and determine implementation success (eg, checklists and criteria). To further strengthen the analysis even further, the included studies were categorized as either including a formal (explicitly integrated body of knowledge) or informal framework (implicit assumptions, beliefs, and views), all following the definitions provided by Nilsen [21].

Fourth, to analyze the literature in relation to the extent to which these findings draw on existing implementation frameworks, a deductive thematic analysis was carried out. This entailed coding the included studies deductively according to the nonadoption, abandonment, scale-up, spread, and sustainability (NASSS) framework for health technologies [5]. This framework suggests a range of subdomains that are relevant to understanding how a health technology might be implemented ranging from a specific health condition to a wider regulatory and sociocultural system. It represents a state-of-the-art structure and has been used in other AI studies [2]. Five reviewers (FG, DT, JN, JR, and PS) interpreted how implementation elements captured in the descriptive themes were related across the NASSS domains. The NASSS framework domains are the condition, technology, value proposition, adopters, organization, wider system, and embedding and adaptation over time. This involved extensive reflection on article findings and how the findings were related to the domains. The process began by deductively assessing the findings in each article and then evaluating their association with NASSS domains. An analytic summary matrix was developed by tabulating the domains for each of the included studies within a table, including identification of new subdomains that did not fit within the existing NASSS framework. Finally, the implementation elements were summarized across the NASSS subdomains and newly identified subdomains to assess the inclusion of elements across the included articles.

## Results

### The Characteristics of Published Literature

The initial search returned 2541 unique articles. We screened all abstracts and eliminated 98.86% (2512/2541) of the papers based on the exclusion criteria. The term implementation framework was widely dispersed across different areas of health care, which resulted in a high number of nonrelated papers. After abstract screening, 29 articles were subjected to full-text review, of which 76% (n=22) of them did not meet the inclusion criteria. Finally, 24% (7/29) of articles were included.

Out of 7 articles, only 2 (29%) of the articles included formal frameworks that directly addressed AI implementation in health care. Owing to the limited number of articles fulfilling the inclusion criteria, articles that partially met the criteria were also included: 14% (1/7) of the articles that included a formal framework addressing issues of ethics and AI (a topic of high importance to real-world AI implementation), and 57% (4/7) of the articles that included descriptions of elements influencing implementation (focusing on physician opinion, patient opinion, and factors influencing the use of AI in emergency departments and surgical settings).

The articles presented heterogeneous study designs. In total, 57% (4/7) of these studies were literature reviews, 29% (2/7) used a qualitative approach, and 71% (1/14) used quantitative survey data. The area of practice and the focus of the selected articles were dispersed. The papers focused on the perceptions of patients and clinicians of AI (n=2), decision-making and decision support systems (n=2), ethical and trustworthy aspects (n=1), benefits and challenges in implementing AI (n=1), and implementation elements to guide AI adoption (n=1). Further characteristics of the included studies are shown in Table 2.

**Table 2:** Characteristics of included papers (n=7).

Study	Country	Study design	Area of practice	Target population	Study focus	Study aims
Beil et al [26]	Germany	Literature review	Intensive care	N/A^a^	Ethical and trustworthy aspects in intensive care	Discuss ethical considerations about AI^b^ for prognostication in intensive care.
Diprose et al [27]	New Zealand	Quantitative study	Primary care	Physicians (n=170)	Perceptions of clinicians to understanding, explain and trust on AI results	Investigate the association between physician understanding of AI outputs, their ability to explain these to patients, and their willingness to trust the AI outputs.
Fernandes et al [28]	Portugal	Literature review	Emergency department	N/A	Intelligent CDSS^c^ for triage	Assess how intelligent CDSS for triage have been contributing to the improvement of quality of care in the ED^d^ as well as to identify the challenges they have been facing regarding implementation.
Loftus et al [29]	United States	Literature review	Operation room	Surgeons	Decision-making in surgeries	Propose that AI models would obviate these weaknesses and be integrated with bedside assessment to augment surgical decision-making.
Nelson et al [30]	United States	Qualitative study	Dermatology clinics	Patients from general dermatology clinics (n=48)	Perception of patients on AI related to skin cancer screening	Explore how patients conceptualize AI and perceive the use of AI for skin cancer screening.
Ngiam et al [31]	Singapore	Literature review	Health care	N/A	Benefits and challenges of AI in oncology	Discuss some of the benefits and challenges of big data and machine learning in health care.
Truong et al [32]	Canada	Qualitative study	Health care	Subject-matter experts in health care (n=8)	Implementation elements to guide AI adoption	Creating an implementation framework to help health care organizations understand the key considerations and guide implementation efforts for AI.

^a^N/A: not applicable.

^b^AI: artificial intelligence.

^c^CDSS: clinical decision support system.

^d^ED: emergency department.

### Quality Assessment

Overall, the quality assessment indicated that 43% (3/7) of articles [26,31,32] met below 30% of the CASP criteria owing to limited descriptions of the methodological procedures. In total, 43% (3/7) of the articles [27-29] met between 50% and 60% of the criteria, whereas 14% (1/7) of articles [30] met 70% of the criteria. The quality scores of each paper in relation to the CASP checklist are provided in the (Multimedia Appendix 2).

### Framework Categories to Implement AI

The analysis identified the use of three primary framework categories: determinant framework, process model, and evaluation framework. Classic theories and implementation theories have not yet been identified. Across the 7 articles, the data analysis indicated that 3 (43%) articles included explicit frameworks: determinant frameworks [32], process models [31], and evaluation frameworks [26]. The remaining articles (4/7, 57%) did not include comprehensive frameworks; instead, elements relevant to implementation could be identified. For example, Beil et al [26] discussed the applicability of ethical constructs in an AI implementation, and Diprose et al [27] explored physician opinion of implementation elements, whereas Nelson et al [30] suggested implementation elements from the patient perspective. These articles did not identify or describe any conceptual or organizational framework to illustrate the relationship between implementation elements. The types of frameworks and elements of each framework (eg, determinants, steps, or aspects) are provided in Table 3. Except for 29% (2/7) of the articles [31,32], most articles lacked a clear description of the elements identified (Table 3).

The data analysis identified the presence of implementation elements in all 7 of the NASSS domains (Table 4). The implementation elements were unequally distributed across the domains and subdomains. A full description of the implementation elements identified in each paper and their relation to NASSS subdomains are provided in the data analysis matrix (Multimedia Appendix 3 [26-32]). Across all papers, technology was the most frequently included domain identified in all articles (7/7, 100%), while embedding and adaptation over time was the less frequently included domain (1/7, 14%). Collectively, the papers explicitly covered 91% (20/22) of the NASSS subdomains; however, there was an uneven distribution between subdomains and between individual articles. Across the 7 articles, the subdomains most frequently identified were *material and features of technology* (7/7, 100%), *types of data generated* (7/7, 100%), and *staff (role and identity*, 6/7, 86%). Less frequently identified subdomains include *scope for adaptation over time* (1/7, 14%) and *extent of change needed to routines* (1/7, 14%).

Among the individual articles, Nelson et al [30] and Beil et al [26] had the highest coverage with 68% (15/22) and 55% (12/22) of the subdomains, respectively. Diprose et al [27] and Fernandes et al [28] covered the fewest subdomains, with only 23% (5/22) of the subdomains. The presence of the elements was not mutually exclusive, as some elements were classified into multiple domains. For example, Beil et al [26] classified the implementation element called explicability in the domains technology, adopters, and wider systems. Similarly, the implementation element human-machine interaction from Ngiam et al [31] was classified in the technology, adopters, organization, and wider system.

In total, 7 new subdomains were identified that did not explicitly fit in the NASSS framework (Table 4). Three elements were classified as belonging to the technology domain: *types of data input*, *dependence/adaptation to the local context*, and *evaluation of effectiveness*. The *types of data inputted* highlight the essential data input into AI algorithms and their dependence on the availability and quality of existing (automated) electronic health record data [29]. *Dependence/adaptation to local contexts* relates to how AI technologies are dependent on other care practices that vary at a local level, such as other technologies in use, data recording methods, and local process of care (eg, patient referral systems) that influence the data input to AI or the ability of local practitioners to trust and act on AI outputs [28]. *Evaluation of effectiveness* makes explicit the need for clinical trials and other validation mechanisms to ascertain the effectiveness, reliability, and trustworthiness of AI algorithms [32]. Under the value proposition domain, we identified the *demand-side value (to population)*, which refers to a medical ethical principle that requires fairness and societal well-being in AI implementation at the population level and considers issues such as data bias and implications for health inequalities within the value proposition of the technology [26]. For domain adopters, we identified *shared decision-making* among patients, professionals, caregivers, and the role of AI as a fourth voice within decision-making processes [30]. Finally, for the domain wider system, we identified *ethics (population equity/discrimination)* and the *role of human oversight*. *Ethics* indicates medical moral principles for beneficence and nonmaleficence at a population level, reflecting the need for formal regulation and consideration of the health equality impact of new technologies at the population level [26,29]. *Human oversight* considers the extent to which it is possible or desirable for technologies to operate with or without human oversight. Together, these underline that AI should not undermine the autonomy of health care professionals nor provoke adverse effects [26,29].

**Table 3:** Descriptions of the frameworks and framework elements in the included articles (n=7).

Study	Explicit framework?	Types of framework^a^ and purpose	Framework elements (stages, determinants, or aspects)	Clarity of element description^b^	Referenced guidance or literature for framework development
Beil et al [26]	Yes	Evaluation framework; ethical AI^c^	Beneficence, nonmaleficence, justice, autonomy, explicability, medical perspective, technical requirements, patient- or family-centered, and system-centered	Partial	European Commission guideline
Diprose et al [27]	No	N/A^d^; elements describe physician opinion of AI	Physician understanding and intended physician behavior, explainability, preferred to explainability methods	Partial	Absent
Fernandes et al [28]	No	N/A; elements describe limitations to develop and implementing AI in ED^e^ triage	Availability of data, the subjectivity of the system, methodologies and modeling techniques, validation, and geography (data from the same geographic area)	Partial	Absent
Loftus et al [29]	No	N/A; elements describe challenges and potential of AI in surgical decision-making	Challenges in surgical decision-making (complexity, values and emotions, time constraints and uncertainty, heuristics and bias), traditional predictive analytics and clinical decision support (decision aids and prognostic scoring systems), AI predictive analytics and augmented decision-making (machine learning, deep learning, and reinforcement learning), implementation (automated electronic health record data, mobile device outputs, and human intuition), challenges to adoption (safety and monitoring, data standardization and technology infrastructure, interpretability, and ethical challenges)	Partial	Absent
Nelson et al [30]	No	N/A; elements describe patient opinion of AI	AI concept, AI benefits, AI risks, AI strengths, AI weaknesses,^f^ AI implementation (symbiosis, credibility, diagnostic tool, setting, and integration into electronic health records. Challenges include malpractice, misunderstanding of AI, and regulations), response to conflict between human and AI clinical decision-making, responsibility for AI accuracy, responsibility for AI data privacy, AI recommendation	Limited	Absent
Ngiam et al [31]	Yes	Process model; AI development and implementation	Clinical problem definition or redefinition, data extraction selection, and refining, data analysis and validation, human-machine interaction, paper trial, prospective clinical trial, medical device registration, and clinical deployment	Explicit	Absent
Truong et al [32]	Yes	Determinant framework; AI implementation	Data quality and quantity, trust, ethics, readiness for change, expertise, buy-in (value creation), regulatory strategy, scalability and evaluation	Explicit	Absent

^a^Type of framework according to the Nilsen taxonomy [21].

^b^Explicit: explicit definition; partial: some discussion, but no explicit definition; limited: only listed construct names, but no definition or discussion is provided.

^c^AI: artificial intelligence.

^d^N/A: not applicable.

^e^ED: emergency department.

^f^Only categories associated with artificial intelligence implementation are shown in full.

**Table 4:** A comparison of elements identified in literature with the nonadoption, abandonment, and challenges to the scale-up, spread, and sustainability (NASSS) framework domains. (n=7).

Condition			Technology		Value proposition		Adopters		Organization		Wider system		Embedding and adaptation over time
**Original NASSS subdomains**													
	Nature of condition^a^ (n=0) Comorbidities, sociocultural influences (n=0)	Material and features of technology (n=7) Types of data generated (n=7) Knowledge needed to use (n=5) Technology supply model (n=2)		Supply-side value (to developer; n=2) Demand-side value (to patient; n=2)		Staff (role and identity; n=6) Patient (simple vs complex input; n=3) Carer (available, nature of input; n=2)		Capacity to innovate (n=1) Readiness for change (n=2) Nature of adoption or funding decision (n=1) Extent of change to new routines (n=1) Work needed to implement change (n=2)		Political or policy (n=2) Regulatory or legal (n=5) Professional (n=2) Sociocultural (n=2)		Scope for adaptation over time (n=1) Organizational resilience (n=1)	
**New NASSS subdomains**													
	Not identified	Types of data inputted (n=3) Dependence on other local processes and practices (n=2) Evaluation of effectiveness (n=3)		Demand-side value (to population; n=1)		Shared decision-making (n=3)		Not identified		Ethics (population equity or discrimination; n=2) Role of human oversight^b^ (n=3)		Not identified	

^a^These elements were not explicitly mentioned in the framework or list of elements, but they were considered in the manuscript (nature of condition, 6 articles; comorbidities and sociocultural influences, 2 articles).

^b^Can be considered across multiple domains.

## Discussion

### Principal Findings

This literature review demonstrates that understanding how to implement AI in health care practice is still in its early stages of development. Although our study search terms identified a large number of articles, only 7 articles were included in the final analysis. Only 29% (2/7) of these articles included formal frameworks that directly addressed AI implementation in health care, and the other 71% (5/7) of the articles provided descriptions of elements influencing such implementation.

The importance of developing knowledge of how to implement AI in health care was highlighted in many of the rejected articles, but despite acknowledging this, the articles provided little or no substance to support their claims, or guidance on how to move forward. A challenge to building knowledge in this field was underscored during the screening process, where many articles mentioned AI but were excluded because they focused on health care technologies unrelated to AI; for example, eHealth and telemedicine. The inappropriate labeling of technologies as AI likely reflects the hype surrounding the AI concept and the tendency to adopt fashionable terms to increase attention, readership, and likeliness of publication [34,35]. This type of misuse of AI terminology creates a murky landscape and ambiguity for researchers attempting to synthesize learning in this emerging field.

Given the recognition of the importance and challenges of AI implementation [2], it was surprising to find that none of the identified articles referred to existing implementation literature in informing data analysis or framework development. Although AI is likely to have specific requirements compared with other health technologies, there is a wealth of literature on implementation challenges and facilitators (eg, Xiang et al [36]) that could inform the AI field and accelerate learning. The only paper informing framework development [26] was the Ethics Guidelines for Trustworthy AI by the European Commission. This suggests that there are attempts within the AI community to reach agreement on key issues relating to the real-world use of AI and that these efforts have not yet been connected with insights from the implementation science community.

To understand the overlap of concerns between AI-specific implementation challenges and more general health technologies, we mapped the elements listed in the 7 papers against the NASSS framework, which was specifically developed to guide the uptake of health technologies [5]. Most elements identified in the papers corresponded to factors within the NASSS framework, suggesting that there is a significant degree of overlap between the concerns of AI implementation and general health technologies, although it should be noted that the general lack of clarification of the element descriptions at times meant they were open to interpretation. The NASSS domain most identified in the 7 AI papers was technology, including the subdomain *material features of the technology* and *types of data generated*, which were included in all 7 papers. The importance of *adopters (staff roles)* was most frequently mentioned (6/7, 86%), followed by *wider system regulatory or legal issues* (5/7, 71%). All other NASSS domains and subdomains were identified in 1 or 2 other papers (except for the condition domain, which did not formally appear in any framework or list of elements, but was nonetheless part of the contextual discussion in most papers). These findings suggest that all NASSS elements are relevant to the implementation of AI in health care, but that awareness and recognition of all these domains are currently low within the AI community. This highlights the value of sharing findings within the AI community, and between AI and implementation science communities, to build a more comprehensive understanding.

A small number of elements identified in the 7 papers did not align with the existing NASSS subdomains. As such, we propose 7 new subdomains that can supplement the NASSS framework. Of the newly identified subdomains, some highlight issues that are likely relevant to all forms of health technologies and may have specific implications within AI. For example, the newly identified subdomain of *shared decision-making* recognizes the need for processes and behaviors that support communication, discussion, and decision-making among staff, patients, and careers (technology adopters), which is likely to be relevant for many data-driven technologies [37-39]. However, in the case of AI, the AI provides a fourth *voice* in the decision-making process that will have particular implications for how such communication is handled in an emotionally sensitive manner, how much weight is given to different opinions and preferences [38,40], and how it could support clinical decision-making without compromising the primary responsibilities and duties of the health care professional for patient care [41]. Similarly, the need for the *evaluation of effectiveness* is important for all health technologies [42,43], but for AI, this may be of particular importance in demonstrating the trustworthiness of data outputs if it is to replace or complement clinical judgment.

The subdomain *role of human oversight* has emerged as a unique implementation feature across multiple domains. The term *human oversight* underlines that AI should not undermine the autonomy of health care professionals nor provoke adverse effects [26,29]. Patients are receptive to the use of AI, yet health care professionals need to have oversight of the AI outcomes and decide when and how to use the information generated from the AI. For example, in particular cases, health care professionals might be able to override the decision made by AI. Unlike *shared decision-making*, which suggests collaborative work between humans and machines for individual patient care, *human oversight* underscores the mandate of health care professionals over AI recommendations as a critical element at the regulatory and system levels. This subdomain has been echoed in the gray literature under analogous terms such as human-in-the-loop, human-on-the-loop, or human-in-command [44].

Other newly identified subdomains appear to be more highly relevant or potentially problematic for AI than for other forms of health technology. For example, understanding the *demand-side* value (population benefit) and *ethics (population equity or discrimination)* appear to be of particular importance to AI, given their dependence or impact on large (population size) data. The possibility of recommending decisions across an entire population entails risks to reinforce systemic biases (eg, White or male), which might unintentionally discriminate minorities and patients with more complex or unusual health conditions. This, in turn, is linked to the increased importance of regulatory and legal systems that oversee the introduction of AI and carefully consider the implications and responsibilities for individuals, professional groups, and governments to ensure the safety, effectiveness, ethics, and equity of new data-driven technologies [40].

### Implications for Practice and Research

Our findings highlight the need to develop an AI-specific implementation framework, drawing on empirical research related to AI implementation efforts, and drawing on existing knowledge and experience within the implementation science community. There is a great, currently unrealized, opportunity to draw on insights from the implementation of science literature to enhance and accelerate the implementation of AI. There is no need to repeat the mistakes or reproduce learning that has already been achieved, and there is an opportunity to use the theoretical and practical insights from others to provide an evidence-based foundation that can accelerate the implementation of AI in health care.

Our findings suggest that the implementation of AI is viewed through a narrow lens, focusing on the design of the technology and its interaction with the immediate user. Lessons from implementation science suggest the need to extend attention to understand how the technology will influence and interact with the context in which it is implemented, including understanding existing processes and practices of care within each local setting, and how systems work at micro, meso, and macro levels to support or hinder technology uptake. Such insights can only be gained from active engagement of relevant stakeholders, frontline staff, patients, and careers, their engagement is essential to understand how new technologies that are based on AI will be received, and how trust can be built to ensure that design is centered on the needs and practical constraints and requirements of the health care system (ie, to produce useful, trusted, relevant, and actionable knowledge). Lessons from implementation science suggest that obtaining this knowledge, using it to inform technology design, and addressing wider implementation issues is time intensive and reliant on good quality relationships between diverse and often conflicting groups of stakeholders. However, time and time again the implementation literature demonstrates the necessity of this work for successful implementation. Harnessing such insights could provide guidance to health care professionals responsible for implementing AI in practice, for decision makers and policy makers to ensure effective implementation plans are in place, and for the AI designers and promotors who need to be aware of the implications of real-world deployment to ensure that AI products are suitable for implementation.

Any AI implementation framework also needs to recognize the heightened and perhaps unique needs and challenges of introducing AI in health care, including meaningful decision support, ethical dilemmas (privacy and consent), transparency, effectiveness, interpretability, and establishing trust in *black box* technologies [2,45,46]. Implementation is further complicated by the symbiotic relationship between AI and the system, the dynamic and interdependent relationship between data input from the health care system to inform the AI, as well as the influence of AI data output on the same system, and the significant technical, analytic, and clinical expertise required to understand and resolve problematic issues. In addition, AI has a greater potential and risk of operating at the population level and the ethical and regulatory requirements to ensure that any such technologies provide equitable population care and safe, effective, and compassionate care at an individual level.

### Methodological Considerations

Although this study was conducted in a structured and systematic manner, only a small number of papers met the inclusion criteria, and these papers were of rather low quality in terms of methodological clarity and rigor, and in the clarity of descriptions and definitions of elements influencing AI implementation. In addition, the included studies that were based on empirical data were conducted only in 3 high-income countries, limiting the generalization of the findings to other contexts. For example, the use of AI in health care in low-income countries is still nascent, and therefore some subdomains of the NASSS framework might be irregularly advanced in this context (eg, legal, regulatory, and social cultural). These characteristics emphasize the importance of reflecting on the findings of parsimony. Together, these limit the reliability and generalizability of the cumulative findings from our analysis and highlight a gap in the literature that requires further empirical and theoretical research.

We chose to use the NASSS framework for deductive analysis of the included papers as it represents one of the most advanced frameworks dedicated to understanding implementation of health care technologies and was informed by extensive empirical research and literature review [2]. Although the use of the NASSS framework has provided a helpful way of making sense of the findings from the AI implementation literature, the use of this framework is illustrative and other frameworks could have been applied to support review and interpretation of results. For example, the NASSS framework adopts a predominantly innovator-centric view of understanding how new technologies can be introduced into a health care system, whereas alternative frameworks provide a more service-centric view in understanding the needs and operating reality of frontline health care services and how new technologies fit with and disturb existing working practices [47]. Further research could be conducted to consider the merits and limitations of different implementation frameworks to provide insights into AI implementation.

Scoping reviews often search for the identification and conceptualization of complex, emergent, or ill-defined concepts. Unlike traditional systematic reviews guided by well-defined constructs, it may be unfeasible to screen and synthesize all relevant literature on an emergent topic [48]. As our purpose was to merely understand what implementation frameworks have been used in the application of AI for health care practices, our efforts to identify all eligible studies were limited in some respects. Moreover, although the results of this review are based on 7 studies, the empirical data were gathered solely from 5 health care–orientated databases. Although a focused sampling technique reduces contextual variations and thus helps provide robust findings, health care professionals working outside health care contexts should consider context-specific variations as they interpret the findings.

### Conclusions

This literature review demonstrates that the research literature on AI implementation in health care lacks theoretical development and is poorly connected to existing implementation frameworks or models developed within implementation science. This means that potential specific challenges around AI implementation are largely unrevealed, and that further empirically based research is needed to provide the knowledge necessary to develop implementation frameworks to guide future implementation of AI in clinical practice.

## Appendix

Multimedia Appendix 1 Health care database search syntax.

Multimedia Appendix 2 Quality appraisal of the selected papers.

Multimedia Appendix 3 Data analysis matrix.

## Abbreviations

AI: artificial intelligence
CASP: Critical Appraisal Skills Programme
MeSH: Medical Subject Headings
NASSS: nonadoption, abandonment, scale-up, spread, and sustainability
PRISMA: Preferred Reporting Items for Systematic Reviews and Meta-Analyses
