# Supplementary material for: Implementation Frameworks for Artificial Intelligence Translation Into Health Care Practice: Scoping Review
Source: J Med Internet Res. 2022 Jan 27;24(1):e32215. doi: 10.2196/32215 (PMC8832266; doi:10.2196/32215)
Supplement: Multimedia Appendix 1 [file jmir_v24i1e32215_app1.docx]

Healthcare database search syntax

| Category | Search term |
| --- | --- |
| Artificial Intelligence | 1. artificial intelligence.mp  2. machine learning.mp  3. 1 or 2 |
| Implementation | 4. implement*.mp  5. implement* framework.mp  6. implement* model.mp  7. implement* theory.mp  8. 4 or 5 or 6 or 7 |
| Search Refinement | 9. 3 and 8  10. limit 9 to date=”2000-01-01 to 2020-03-31”  11. limit 10 to English language  12. limit 11 to Human  13. remove duplicates form 16 |
