# Supplementary material for: Implementation Frameworks for Artificial Intelligence Translation Into Health Care Practice: Scoping Review
Source: J Med Internet Res. 2022 Jan 27;24(1):e32215. doi: 10.2196/32215 (PMC8832266; doi:10.2196/32215)
Supplement: Multimedia Appendix 2 [file jmir_v24i1e32215_app2.docx]

Quality appraisal of the selected papers

| Author(s), Year | Question 1 | Question 2 | Question 3 | Question 4 | Question 5 | Question 6 | Question 7 | Question 8 | Question 9 | Question 10 | Total of Yes |
| --- | --- | --- | --- | --- | --- | --- | --- | --- | --- | --- | --- |
| Beil et al., 2019 | No | No | No | No | Can’t tell | Can’t tell | No | Yes | Can’t tell | Yes | 2 |
| Diprose et al., 2020 | Yes | Yes | Can’t tell | No | Yes | Yes | No | Yes | Can’t tell | Yes | 6 |
| Fernandes et al., 2020 | No | Yes | Yes | No | Yes | Yes | Can´t tell | Yes | Can’t tell | Can´t tell | 5 |
| Loftus et al., 2020 | Yes | Yes | Yes | No | Yes | Yes | No | Yes | Can´t tell | Can´t tell | 4 |
| Nelson et al., 2020 | Yes | Yes | Yes | Yes | Yes | Yes | Can´t tell | Yes | No | No | 7 |
| Ngiam et al., 2019 | No | No | Can´t tell | No | Yes | No | No | Yes | Can’t tell | Yes | 3 |
| Truong et al., 2019 | No | No | No | No | No | No | No | No | No | No | 0 |

List the questions suggested to literature review articles

1. Did the review address a clearly focused question?

2. Did the authors look for the right type of papers?

3. Do you think all the important, relevant studies were included?

4. Did the review’s authors do enough to assess quality of the included studies?

5. If the results of the review have been combined, was it reasonable to do so?

6. What are the overall results of the review?

7. How precise are the results?

8. Can the results be applied to the local population?

9. Were all important outcomes considered?

10. Are the benefits worth the harms and costs?
