# Supplementary material for: Implementation Frameworks for Artificial Intelligence Translation Into Health Care Practice: Scoping Review
Source: J Med Internet Res. 2022 Jan 27;24(1):e32215. doi: 10.2196/32215 (PMC8832266; doi:10.2196/32215)
Supplement: Multimedia Appendix 3 [file jmir_v24i1e32215_app3.docx]

Data analysis matrix

| **# Element** | **Authors** | **Framework element** |  | **A - Condition** | | **B - Technology** | | | | **C - Value proposition** | | **D - Adopters** | | | **E - Organization** | | | | | **F - Wider system** | | | | **G - Embedding and adaptation over time** | | **Emergent sub-domains** |
| --- | --- | --- | --- | --- | --- | --- | --- | --- | --- | --- | --- | --- | --- | --- | --- | --- | --- | --- | --- | --- | --- | --- | --- | --- | --- | --- |
|  |  |  | **Number of articles** | **n = 6** | **n = 2** | **n = 7** | **n = 5** | **n = 7** | **n = 2** | **n = 2** | **n = 2** | **n = 6** | **n = 3** | **n = 2** | **n = 1** | **n = 2** | **n = 1** | **n = 1** | **n = 2** | **n = 2** | **n = 5** | **n = 2** | **n = 2** | **n = 1** | **n = 1** |  |
|  |  |  | **Total number of elements** | **6** | **2** | **36** | **9** | **34** | **5** | **2** | **44** | **46** | **31** | **2** | **1** | **2** | **1** | **1** | **3** | **4** | **35** | **10** | **2** | **1** | **1** |  |
|  |  |  | **Representative quote from articles** | 1 - Nature of condition | 2 - Comorbidities, socio-cultural influences | 1 - Material & features of the technology | 3 - Knowledge needed to use | 2 - Types of data generated (accuracy, data acceptance, trust) | 4 - Technology supply model | 1 - Supply-side value (to developer) | 2 - Demand-side value (to patient) | 1- Staff (role, identity) | 2- Patient (simple v complex input) | 3 - Carers (availabe, nature of input) | 1 - Capacity to innovate (leadership etc) | 2 - Readiness for this change or technology | 3 - Nature of adoption /funding decision | 4 -Extent of change needed to routines | 5 - Work needed to implement change | 1- Political / policy | 2 - Regulatory / legal | 3 - Professional | 4 - Socio-cultural | 1 - Scope for adaptation over time | 2 - Organisational resilience |  |
| 1 | Beil et al., 2019 | Condition | Prognosticating the course of critical illnesses and predicting the impact of interventions are major pillars of decision-making in intensive care. A substantial number of inviduals in intensive care, present more complex disorders. | 1 |  |  |  |  |  |  |  |  |  |  |  |  |  |  |  |  |  |  |  |  |  |  |
| 2 | Beil et al., 2019 | Medical | What is the purpose? What are the consequences, risks and alternatives? |  |  |  |  |  |  |  | 1 |  |  |  |  |  |  |  |  |  |  |  |  |  |  |  |
| 3 | Beil et al., 2019 | Technical | What is the model? What were the training data? Can the uncertainty be qualified? |  |  | 1 |  |  |  |  |  |  |  |  |  |  |  |  |  |  |  |  |  |  |  |  |
| 4 | Beil et al., 2019 | Patients-centered | What are the individual goals? Who is responsible? Is the situation understood? Is there informed consent? |  |  |  |  |  |  |  |  |  | 1 |  |  |  |  |  |  |  |  |  |  |  |  |  |
| 5 | Beil et al., 2019 | System-centered | What is the system oversight? How are different conclusions reconciled? |  |  |  | 1 |  | 1 |  |  | 1 |  |  |  |  |  |  |  |  | 1 |  |  |  |  | Role of human oversight |
| 6 | Beil et al., 2019 | Beneficence | Positive value of AI. requirement for implementation: Data stewardship, accountability |  |  |  | 1 | 1 |  |  | 1 |  |  |  |  |  |  |  |  |  | 1 |  |  |  |  | Ethics (population equity / discrimination) |
| 7 | Beil et al., 2019 | Non-maleficence | Risk of false hope, false despair, uncertainty. Requirement for implementation: Technical robustness (accuracy and uncertainty) |  |  |  | 1 | 1 |  |  | 1 |  |  |  |  |  |  |  |  |  | 1 |  |  |  |  | Ethics (population equity / discrimination) |
| 8 | Beil et al., 2019 | Justice | Distribution of resources within a society and non-discimrination of individuals. requirement for implementation: Fairness, societal wellbeing |  |  |  |  |  |  |  | 1 |  |  |  |  |  |  |  |  | 1 | 1 |  | 1 |  |  | Deman-side value (to population) |
| 9 | Beil et al., 2019 | Autonomy | Patient autonomy - capacity for self-determination balanced with requests for potentially inappropriate treatment. requirement for implementation: Human agency and oversight. |  |  |  |  |  |  |  |  | 1 | 1 |  |  |  |  |  |  |  | 1 | 1 |  |  |  | Role of human oversight |
| 10 | Beil et al., 2019 | Explicability | Transparency of models in producing outputs based on specific inputs. Trust and ability to interact with models. Moral and legal accountability. requirement for implementation: transparency. |  |  | 1 |  | 1 |  |  |  | 1 | 1 |  |  |  |  |  |  |  | 1 |  |  |  |  |  |
| 11 | Diprose et al. 2020 | Condition | No specific intervention. Diagnostic dilemma related to pulmonary embolism. The rationale for this clinical scenario was that pulmonary embolism is a disease encountered in community, medical, and surgical settings and is potentially life-threatening, so decision making around diagnosis and management is critical. | 1 |  |  |  |  |  |  |  |  |  |  |  |  |  |  |  |  |  |  |  |  |  |  |
| 12 | Diprose et al. 2020 | Physician understanding and intended physician behaviour (trust) | Our study also demonstrated this trend, with no physician reporting that they would follow the ML output if they rated their understanding as “not at all.” Whilst this evidence base is small and heterogeneous, it suggests an association between physician understanding of ML outputs and intended physician behavior. |  |  |  | 1 |  |  |  |  | 1 |  |  |  |  |  |  |  |  |  |  |  |  |  |  |
| 13 | Diprose et al. 2020 | Explanability | Providing more information about the logic underlying a ML output for the physician to verify may reduce the incidence of these errors and, incidentally, may also address concerns of physician deskilling from automation complacency |  |  | 1 | 1 | 1 |  |  |  | 1 |  |  |  |  |  |  |  |  |  |  |  |  |  |  |
| 14 | Diprose et al. 2020 | Preferred explainability methods | Among the 88% physicians who preferred a model-agnostic explanation, 62% preferred a local explanation over a global explanation. |  |  | 1 | 1 | 1 |  |  |  | 1 |  |  |  |  |  |  |  |  |  |  |  |  |  |  |
| 15 | Fernandes et al., 2020 | Condition | There could be other important factors missed in the model, like the presenting symptoms, the vital signs, and functional and socio-economic status of patients. |  | 1 |  |  |  |  |  |  |  |  |  |  |  |  |  |  |  |  |  |  |  |  |  |
| 16 | Fernandes et al., 2020 | Condition | There may be critical patients presenting symptoms not easily recognized as indicators of criticality. To develop models to addists in the prioritiztion of patients, according to their acuity level at the triage. | 1 |  |  |  |  |  |  |  |  |  |  |  |  |  |  |  |  |  |  |  |  |  |  |
| 17 | Fernandes et al., 2020 | Subjectivity of the system | Another limitation that was highlighted consisted in the subjectivity of the triage CDSS (clinical decision support system), since these are reliant on the operator. Thus, we stress that it is of foremost importance to assess the receptivity of health professionals to the use of the CDSS and that they receive adequate training. |  |  |  | 1 |  |  |  |  |  |  |  |  |  |  |  |  |  |  |  |  |  |  |  |
| 18 | Fernandes et al., 2020 | Availiability of data | 1) " working with larger clinical datasets in order to apply intelligent techniques and extract knowledge", 2) "could be other important factors missed in the model, like the presenting symptoms, the vital signs, and functional or socio-economic status of patients. This fact may have limited the discrimination and validation power of the model." |  |  | 1 |  |  |  |  |  |  |  |  |  |  |  |  |  |  |  |  |  |  |  | Types of data inputted |
| 19 | Fernandes et al., 2020 | Methodologies and modelling techniques | To identify an association of their DSS with access block (to inpatient beds) and patient mortality and also its possible use to predict the access block (to inpatient beds)", "their DSS should be compared to the rates of undertriage and overtriage", and "show that the DSS significantly outperformed triage nurses’ predictions in a direct comparison, and to demonstrate the extent to which incorporation of the DSS into clinical practice actually improved care or use of resources. |  |  | 1 |  |  |  |  |  |  |  |  |  |  |  |  |  |  |  |  |  |  |  |  |
| 20 | Fernandes et al., 2020 | Validation | Half of the studies lacked the validation phase of their CDSS. In the study bootstrapping was used for internal validation." cross validation was performed. "In [29], both internal and external validation were performed, first in a tertiary referral hospital and second in an urban community hospital." |  |  | 1 |  |  |  |  |  |  |  |  |  |  |  |  |  |  |  |  |  |  |  | Evaluation of effectiveness |
| 21 | Fernandes et al., 2020 | Geography | The authors recognized that the main limitation of the study was that, although it used data from different units, the hospitals were all in the same geographic region. This meant that they shared similar working practices, data recording methods, tertiary referral services and patient demographics. |  |  | 1 |  |  |  |  |  |  |  |  | 1 |  |  |  |  |  |  |  |  |  |  | Dependence on other local processes |
| 22 | Loftus et al., 2020 | Condition | Surgical patients, designed to augment surgical decision making. Facing uncertainty, owing to missing or incomplete data. Urgent and emergent condition. Most disease are not driven by a single physiological parameter | 1 |  |  |  |  |  |  |  |  |  |  |  |  |  |  |  |  |  |  |  |  |  |  |
| 23 | Loftus et al., 2020 | Challenges to Adoption - Interpretability | Diligent clinicians and informed patients will want to know why a computer program made a certain prediction or recommendation. Several techniques address this challenge, including attention mechanisms. |  |  |  | 1 |  |  |  |  | 1 |  |  |  |  |  |  |  |  |  |  |  |  |  |  |
| 24 | Loftus et al., 2020 | Challenges to Adoption - Safety and monitoring | If model inputs are flawed or model outputs are not carefully monitored by data scientists and interpreted by astute clinicians, many patients could be harmed in a short time frame. Artificial intelligence models trained on erroneous or misrepresentative data are likely to obscure the truth. Prior to clinical implementation, machine and deep learning models must be rigorously analyzed in a retrospective fashion and externally validated to ensure generalizability. |  |  | 1 |  |  |  |  |  |  |  |  |  |  |  |  |  |  | 1 |  |  | 1 |  |  |
| 25 | Loftus et al., 2020 | Challenges to Adoption - Data standardization and technology infrastructure | To produce models that may be integrated with any EHR in any setting, data must be standardized. The Fast Healthcare Interoperability Resources framework establishes standards for health information exchange using a set of universal components assembled into systems that facilitate data sharing across EHRs and cloud-based communications. |  |  | 1 |  |  |  |  |  |  |  |  |  |  |  |  |  |  |  |  |  |  |  | Dependence on other local processes |
| 26 | Loftus et al., 2020 | Challenges to Adoption - Ethical challenges | When algorithms are trained on data sets that are influenced by bias, algorithm outputs will likely reflect similar bias. In 1 prominent example, a model designed to augment judicial decision-making by predicting the likelihood of crime recidivism demonstrated predilection for racial/ethnic discrimination. |  |  | 1 |  |  |  |  |  |  |  |  |  |  |  |  |  |  | 1 |  |  |  |  |  |
| 27 | Loftus et al., 2020 | Implementation - Automated Electronic Health Record Data |  |  |  | 1 |  |  | 1 |  |  |  |  |  |  |  |  |  |  |  |  |  |  |  |  | Types of data inputted |
| 28 | Loftus et al., 2020 | Implementation - Mobile Device Outputs |  |  |  | 1 |  | 1 | 1 |  |  |  |  |  |  |  |  |  |  |  |  |  |  |  |  |  |
| 29 | Loftus et al., 2020 | Challenges in Surgical Decision-making, complexity |  |  |  |  |  |  |  |  |  | 1 |  |  |  |  |  |  |  |  |  |  |  |  |  |  |
| 30 | Loftus et al., 2020 | Challenges in Surgical Decision-making, Values and emotions |  |  |  |  |  |  |  |  |  | 1 | 1 | 1 |  |  |  |  |  |  |  |  |  |  |  | Shared decision making |
| 31 | Loftus et al., 2020 | Challenges in Surgical Decision-making, Time constraints and uncertainty |  |  |  |  |  |  |  |  |  | 1 |  |  |  |  |  |  |  |  |  |  |  |  |  | Dependence on other local processes |
| 32 | Loftus et al., 2020 | Challenges in Surgical Decision-making, Heuristics and Bias |  |  |  |  |  |  |  |  |  | 1 |  |  |  |  |  |  |  |  |  |  |  |  |  |  |
| 33 | Loftus et al., 2020 | Traditional Predictive Analytics and Clinical Decision Support, Decisions Aids |  |  |  | 1 |  | 1 |  |  |  |  |  |  |  |  |  |  |  |  |  |  |  |  |  |  |
| 34 | Loftus et al., 2020 | Tradicional Predictive Analytics and Clinical Decision Support, Prognostic Scoring Systems |  |  |  | 1 |  | 1 |  |  |  |  |  |  |  |  |  |  |  |  |  |  |  |  |  |  |
| 35 | Loftus et al., 2020 | Artificial Intelligence Predictive Analytics and Augmented Decision-making, Machine Learning |  |  |  | 1 |  | 1 |  |  |  |  |  |  |  |  |  |  |  |  |  |  |  |  |  |  |
| 36 | Loftus et al., 2020 | Artificial Intelligence Predictive Analytics and Augmented Decision-making, Deep Learning |  |  |  | 1 |  | 1 |  |  |  |  |  |  |  |  |  |  |  |  |  |  |  |  |  | Shared decision making |
| 37 | Loftus et al., 2020 | Artificial Intelligence Predictive Analytics and Augmented Decision-making, Reinforcement Learning |  |  |  | 1 |  | 1 |  |  |  |  |  |  |  |  |  |  |  |  |  |  |  |  |  |  |
| 38 | Nelson et al., 2020 | Condition | The demographic characteristics of our patient may limit generalizability to other study populations. Future studies are essential to elucidate perspectives of patients with diverse racial, ethnic, and socioeconomic backgrounds and with varying levels of education and access to dermatologic care. |  | 1 |  |  |  |  |  |  |  |  |  |  |  |  |  |  |  |  |  |  |  |  |  |
| 39 | Nelson et al., 2020 | Condition | Dermatology to classify skin lesions. Skin cancer screening. Hypothetical scenario, | 1 |  |  |  |  |  |  |  |  |  |  |  |  |  |  |  |  |  |  |  |  |  |  |
| 40 | Nelson et al., 2020 | AI - Implementation, Symbiosis | 94% expressed the importance of symbiosis between humans and AI. The term man-computer symbiosis was first used by Licklider et al. to describe a form of teamwork in which humans provide strategic input while computers provide depth of analysis. |  |  |  |  |  | 1 |  |  | 1 |  |  |  |  |  |  |  |  |  |  |  |  |  | Shared decision making |
| 41 | Nelson et al., 2020 | AI - Implementation,Credibility |  |  |  |  |  | 1 |  |  |  | 1 |  |  |  |  |  |  |  |  |  |  |  |  |  |  |
| 42 | Nelson et al., 2020 | AI - Implementation,Challenges include malpractice |  |  |  |  |  |  |  |  |  | 1 |  |  |  |  |  |  |  |  |  |  |  |  |  |  |
| 43 | Nelson et al., 2020 | AI - Implementation,Challenges include misunderstanding of AI |  |  |  |  |  |  |  |  |  | 1 |  |  |  |  |  |  |  |  |  |  |  |  |  |  |
| 44 | Nelson et al., 2020 | AI - Implementation,Challenges include regulations | Unless you can guarantee under penalty of being sued out of existence that [AI] will be 100% accurate all the time, you need to…soften the presumption of accuracy. |  |  |  |  |  |  |  |  |  |  |  |  |  |  |  |  |  | 1 |  |  |  |  |  |
| 45 | Nelson et al., 2020 | AI - Implementation,Diagnostic tool | Maybe if a mole was changing, there would be a way to track that… If the doctor said, this is a watch and wait…maybe [AI] could…take picturesof it and feed that information to the doctor. |  |  | 1 |  | 1 |  |  |  |  |  |  |  |  |  |  |  |  |  |  |  |  |  |  |
| 46 | Nelson et al., 2020 | AI - Implementation, Integration into electronic health records | However well [AI] gets integrated with Partners…would be fantastic. |  |  |  |  |  | 1 |  |  |  |  |  |  |  |  |  |  |  |  |  |  |  |  |  |
| 47 | Nelson et al., 2020 | AI - Implementation,Setting | (subcodes: health care institution: academic vs private; patient: age, intelligence, medical history) |  |  |  |  | 1 |  |  |  |  |  |  |  | 1 |  |  |  |  |  |  |  |  |  |  |
| 48 | Nelson et al., 2020 | AI - Concept, Cognition | (subcodes: game playing, human cognitive support, intelligence superior to human, self-learning) |  |  | 1 |  |  |  |  |  |  |  |  |  |  |  |  |  |  |  |  |  |  |  |  |
| 49 | Nelson et al., 2020 | AI - Concept, Machine | (subcodes: computer, Google, inhuman, robot) |  |  | 1 |  |  |  |  |  |  |  |  |  |  |  |  |  |  |  |  |  |  |  |  |
| 50 | Nelson et al., 2020 | AI - Concept, Modernity |  |  |  | 1 |  |  |  |  |  |  |  |  |  |  |  |  |  |  |  |  |  |  |  |  |
| 51 | Nelson et al., 2020 | AI - Concept, NO AI concept |  |  |  |  |  |  |  |  |  |  |  |  |  |  |  |  |  |  |  |  |  |  |  |  |
| 52 | Nelson et al., 2020 | AI - Concept, Science fiction | (subcode: outer space) |  |  | 1 |  |  |  |  |  |  |  |  |  |  |  |  |  |  |  |  |  |  |  |  |
| 53 | Nelson et al., 2020 | AI - Concept, Specialized vs generalized |  |  |  | 1 |  |  |  |  |  |  |  |  |  |  |  |  |  |  |  |  |  |  |  |  |
| 54 | Nelson et al., 2020 | AI - Benefits, Increase diagnostic speed | (subcodes: early skin cancer detection, lifesaving potential) |  |  |  |  |  |  |  | 1 |  |  |  |  |  |  |  |  |  |  |  |  |  |  |  |
| 55 | Nelson et al., 2020 | AI - Benefits, Increase health care access | (subcodes: increase labor efficiency, increase time for physician-patient interaction, remote diagnosis, unburden the health care system) |  |  |  |  |  |  |  | 1 |  |  |  |  |  |  |  |  |  |  |  |  |  |  |  |
| 56 | Nelson et al., 2020 | AI - Benefits, Reduce health care cost |  |  |  |  |  |  |  |  | 1 |  |  |  |  |  |  |  |  |  |  |  |  |  |  |  |
| 57 | Nelson et al., 2020 | AI - Benefits, Reduce patient anxiety |  |  |  |  |  |  |  |  | 1 |  |  |  |  |  |  |  |  |  |  |  |  |  |  |  |
| 58 | Nelson et al., 2020 | AI - Benefits, Increase triage efficiency |  |  |  |  |  |  |  |  | 1 |  |  |  |  |  |  |  |  |  |  |  |  |  |  |  |
| 59 | Nelson et al., 2020 | AI - Benefits, Reduce uncessary biopsies |  |  |  |  |  |  |  |  | 1 |  |  |  |  |  |  |  |  |  |  |  |  |  |  |  |
| 60 | Nelson et al., 2020 | AI - Benefits, Increase patient self-advocacy |  |  |  |  |  |  |  |  | 1 |  |  |  |  |  |  |  |  |  |  |  |  |  |  |  |
| 61 | Nelson et al., 2020 | AI - Benefits, Stimulate technology |  |  |  |  |  |  |  |  | 1 |  |  |  |  |  |  |  |  |  |  |  |  |  |  |  |
| 62 | Nelson et al., 2020 | AI - Benefits, Patient gain of privacy |  |  |  |  |  |  |  |  | 1 |  |  |  |  |  |  |  |  |  |  |  |  |  |  |  |
| 63 | Nelson et al., 2020 | AI - Risks, Increase patient anxiety |  |  |  |  |  |  |  |  | 1 |  |  |  |  |  |  |  |  |  |  |  |  |  |  |  |
| 64 | Nelson et al., 2020 | AI - Risks, Human loss of social interaction |  |  |  |  |  |  |  |  | 1 |  |  |  |  |  |  |  |  |  |  |  |  |  |  |  |
| 65 | Nelson et al., 2020 | AI - Risks, Patient loss of privacy |  |  |  |  |  |  |  |  | 1 |  |  |  |  |  |  |  |  |  |  |  |  |  |  |  |
| 66 | Nelson et al., 2020 | AI - Risks, Patient loss of follow-up |  |  |  |  |  |  |  |  | 1 |  |  |  |  |  |  |  |  |  |  |  |  |  |  |  |
| 67 | Nelson et al., 2020 | AI - Risks, Nefarious use of AI | (subcodes: humans as “guinea pigs” for testing AI, human commoditization) |  |  |  |  |  |  |  | 1 |  |  |  |  |  |  |  |  |  | 1 |  |  |  |  | Ethics (population equity / discrimination) |
| 68 | Nelson et al., 2020 | AI - Risks, Human deskilling | (subcode: increase human dependence on technology) |  |  |  |  |  |  |  |  | 1 |  |  |  |  |  |  |  |  |  |  |  |  |  |  |
| 69 | Nelson et al., 2020 | AI - Risks, Human loss of control over AI |  |  |  |  |  |  |  |  | 1 | 1 |  |  |  |  |  |  |  |  | 1 |  |  |  |  | Ethics (population equity / discrimination) |
| 70 | Nelson et al., 2020 | AI - Risks, human jobs |  |  |  |  |  |  |  |  |  | 1 |  |  |  |  |  |  |  |  |  |  |  |  |  |  |
| 71 | Nelson et al., 2020 | AI - Risks, Reduce health care access | (subcodes: reduce labor efficiency, burden the health care system) |  |  |  |  |  |  |  | 1 |  |  |  |  |  |  |  |  |  |  |  |  |  |  |  |
| 72 | Nelson et al., 2020 | AI - Risks, Risks of patient physical harm due to use of AI technology |  |  |  |  |  |  |  |  | 1 |  |  |  |  |  |  |  |  |  |  |  |  |  |  |  |
| 73 | Nelson et al., 2020 | AI - Risks, Increase health care cost |  |  |  |  |  |  |  |  | 1 |  |  |  |  |  | 1 |  |  |  |  |  |  |  |  |  |
| 74 | Nelson et al., 2020 | AI - Risks, Increase health care disparities | (subcode: increase disparity in access to AI) |  |  |  |  |  |  |  | 1 |  |  |  |  |  |  |  |  | 1 | 1 |  | 1 |  |  | Ethics (population equity / discrimination) |
| 75 | Nelson et al., 2020 | AI - Risks, Reduce diagnostic speed |  |  |  |  |  |  |  |  | 1 |  |  |  |  |  |  |  |  |  |  |  |  |  |  |  |
| 76 | Nelson et al., 2020 | AI - Risks, Reduce trust in health care professionals |  |  |  |  |  |  |  |  | 1 | 1 | 1 |  |  |  |  |  |  |  |  |  |  |  |  |  |
| 77 | Nelson et al., 2020 | AI - Risks, Risks of patient physical harm due to self-treatment |  |  |  |  |  |  |  |  | 1 |  | 1 |  |  |  |  |  |  |  |  |  |  |  |  |  |
| 78 | Nelson et al., 2020 | AI - Strenghts, More accurate diagnosis | (subcodes: ability to draw on more data or experience than humans, ability to learn and evolve, ability to share data) |  |  | 1 |  | 1 |  |  | 1 |  |  |  |  |  |  |  |  |  |  |  |  |  |  |  |
| 79 | Nelson et al., 2020 | AI - Strenghts, Patient activiation | (activate patient to seek out health information, activate patient to seek out health care) |  |  |  |  |  |  |  |  |  | 1 |  |  |  |  |  |  |  |  |  |  |  |  |  |
| 80 | Nelson et al., 2020 | AI - Strenghts, More convenient diagnosis |  |  |  |  |  |  |  |  | 1 | 1 | 1 |  |  |  |  |  |  |  |  |  |  |  |  |  |
| 81 | Nelson et al., 2020 | AI - Strenghts, More consistent diagnosis |  |  |  |  |  |  |  |  | 1 | 1 | 1 |  |  |  |  |  |  |  |  |  |  |  |  |  |
| 82 | Nelson et al., 2020 | AI - Strengths, More objective diagnosis | (subcode: human distraction, human emotion, human impairment) |  |  |  |  |  |  |  | 1 | 1 | 1 |  |  |  |  |  |  |  |  |  |  |  |  |  |
| 83 | Nelson et al., 2020 | AI - Strenghts, Patient education |  |  |  |  |  |  |  |  | 1 |  | 1 |  |  |  |  |  |  |  |  |  |  |  |  |  |
| 84 | Nelson et al., 2020 | AI - Weaknesses, Less accurated diagnosis | (subcodes: false-negative, false-positive, inaccurate or limited training set, lack of context [eg, patient history], lack of physical examination [(eg, palpation, view from multiple angles], operator dependence) |  |  |  |  |  |  |  | 1 | 1 | 1 |  |  |  |  |  |  |  |  |  |  |  |  |  |
| 85 | Nelson et al., 2020 | AI - Weaknesses, Lack of verbal communication | (subcodes: inability to answer patient follow-up questions, inability to discuss treatment options with patient, inability to educate patient, inability to reassure patient) |  |  |  |  |  |  |  | 1 | 1 | 1 |  |  |  |  |  |  |  |  |  |  |  |  | Shared decision making |
| 86 | Nelson et al., 2020 | AI - Weaknesses, Lack of emotion | (subcodes: lack of compassion, lack of empathy) |  |  |  |  |  |  |  | 1 | 1 | 1 |  |  |  |  |  |  |  |  |  |  |  |  | Shared decision making |
| 87 | Nelson et al., 2020 | AI - Weaknesses, Lack of nonverbal communication | (subcodes: lack of emotion perception, lack of eye contact, lack of physical contact) |  |  |  |  |  |  |  | 1 | 1 | 1 |  |  |  |  |  |  |  |  |  |  |  |  |  |
| 88 | Nelson et al., 2020 | AI - Weaknesses, Lack of creativity |  |  |  |  |  |  |  |  | 1 | 1 | 1 |  |  |  |  |  |  |  |  |  |  |  |  |  |
| 89 | Nelson et al., 2020 | AI - Weaknesses, Lack of social contract between AI and patient | (subcodes: lack of patient accountability) |  |  |  |  |  |  |  | 1 |  | 1 |  |  |  |  |  |  |  |  |  |  |  |  |  |
| 90 | Nelson et al., 2020 | AI - Weaknesses, Lack of total body skin examination |  |  |  |  |  |  |  |  | 1 | 1 | 1 |  |  |  |  |  |  |  |  |  |  |  |  |  |
| 91 | Nelson et al., 2020 | AI - Weaknesses, Limited to visual inspection |  |  |  |  |  |  |  |  | 1 | 1 | 1 |  |  |  |  |  |  |  |  |  |  |  |  |  |
| 92 | Nelson et al., 2020 | AI - Weaknesses, Uniformity restricts patient choice of health care professional |  |  |  |  |  |  |  |  | 1 | 1 | 1 |  |  |  |  |  |  |  |  |  |  |  |  |  |
| 93 | Nelson et al., 2020 | Response to Conflict Between Human and AI Clinical Decision-Making, seek a biopsy |  |  |  |  |  |  |  |  |  | 1 | 1 |  |  |  |  |  |  |  | 1 | 1 |  |  |  |  |
| 94 | Nelson et al., 2020 | Response to Conflict Between Human and AI Clinical Decision-Making, Trust the physician |  |  |  |  |  |  |  |  |  | 1 | 1 |  |  |  |  |  |  |  | 1 | 1 |  |  |  |  |
| 95 | Nelson et al., 2020 | Response to Conflict Between Human and AI Clinical Decision-Making, Seek an opnion from another physician |  |  |  |  |  |  |  |  |  | 1 | 1 |  |  |  |  |  |  |  | 1 | 1 |  |  |  |  |
| 96 | Nelson et al., 2020 | Response to Conflict Between Human and AI Clinical Decision-Making, Seek longitudinal follow-up from the same physician |  |  |  |  |  |  |  |  |  | 1 | 1 |  |  |  |  |  |  |  |  |  |  |  |  |  |
| 97 | Nelson et al., 2020 | Response to Conflict Between Human and AI Clinical Decision-Making, Discontinue use of AI |  |  |  |  |  |  |  |  |  | 1 | 1 |  |  |  |  |  |  |  |  |  |  |  |  |  |
| 98 | Nelson et al., 2020 | Response to Conflict Between Human and AI Clinical Decision-Making, seek another opinion from another AI tool |  |  |  |  |  |  |  |  |  | 1 | 1 |  |  |  |  |  |  |  |  |  |  |  |  |  |
| 99 | Nelson et al., 2020 | Response to Conflict Between Human and AI Clinical Decision-Making, Seek longitudinal follow-up from the same AI tool |  |  |  |  |  |  |  |  |  | 1 | 1 |  |  |  |  |  |  |  |  |  |  |  |  |  |
| 100 | Nelson et al., 2020 | Response to Conflict Between Human and AI Clinical Decision-Making, seek an opinion from family member or friend |  |  |  |  |  |  |  |  |  | 1 | 1 | 1 |  |  |  |  |  |  |  |  |  |  |  | Shared decision making |
| 101 | Nelson et al., 2020 | Responsability for AI Accuracy, Technology company |  |  |  |  |  | 1 |  |  |  |  |  |  |  |  |  |  |  |  | 1 |  |  |  |  |  |
| 102 | Nelson et al., 2020 | Responsability for AI Accuracy, physician |  |  |  |  |  | 1 |  |  |  | 1 |  |  |  |  |  |  |  |  | 1 | 1 |  |  |  |  |
| 103 | Nelson et al., 2020 | Responsability for AI Accuracy, Collective |  |  |  |  |  | 1 |  |  |  |  |  |  |  |  |  |  |  |  | 1 | 1 |  |  |  |  |
| 104 | Nelson et al., 2020 | Responsability for AI Accuracy, Health care institution |  |  |  |  |  | 1 |  |  |  |  |  |  |  |  |  |  |  |  | 1 | 1 |  |  |  |  |
| 105 | Nelson et al., 2020 | Responsability for AI Accuracy, Government |  |  |  |  |  | 1 |  |  |  |  |  |  |  |  |  |  |  | 1 | 1 |  |  |  |  |  |
| 106 | Nelson et al., 2020 | Responsability for AI Accuracy, Patient |  |  |  |  |  | 1 |  |  |  |  | 1 |  |  |  |  |  |  |  |  |  |  |  |  |  |
| 107 | Nelson et al., 2020 | Responsability for AI Accuracy, Organized dermatology |  |  |  |  |  | 1 |  |  |  |  |  |  |  |  |  |  |  |  | 1 | 1 |  |  |  |  |
| 108 | Nelson et al., 2020 | Responsability for AI Accuracy, unsure |  |  |  |  |  |  |  |  |  |  |  |  |  |  |  |  |  |  | 1 |  |  |  |  |  |
| 109 | Nelson et al., 2020 | Responsibility for AI Data Privacy, health care institution |  |  |  | 1 |  | 1 |  |  |  |  |  |  |  |  |  |  |  |  | 1 |  |  |  |  |  |
| 110 | Nelson et al., 2020 | Responsibility for AI Data Privacy, Technology company |  |  |  | 1 |  | 1 |  |  |  |  |  |  |  |  |  |  |  |  | 1 |  |  |  |  |  |
| 111 | Nelson et al., 2020 | Responsibility for AI Data Privacy, Government |  |  |  | 1 |  | 1 |  |  |  |  |  |  |  |  |  |  |  | 1 | 1 |  |  |  |  |  |
| 112 | Nelson et al., 2020 | Responsibility for AI Data Privacy, Database manager |  |  |  | 1 |  | 1 |  |  |  |  |  |  |  |  |  |  |  |  | 1 |  |  |  |  |  |
| 113 | Nelson et al., 2020 | Responsibility for AI Data Privacy, Physician |  |  |  | 1 |  | 1 |  |  |  | 1 |  |  |  |  |  |  |  |  | 1 | 1 |  |  |  |  |
| 114 | Nelson et al., 2020 | Responsibility for AI Data Privacy, Unsure |  |  |  |  |  |  |  |  |  |  |  |  |  |  |  |  |  |  | 1 |  |  |  |  |  |
| 115 | Nelson et al., 2020 | Responsibility for AI Data Privacy, Collective |  |  |  | 1 |  | 1 |  |  |  | 1 |  |  |  |  |  |  |  |  | 1 | 1 |  |  |  |  |
| 116 | Nelson et al., 2020 | Responsibility for AI Data Privacy, Patient |  |  |  | 1 |  | 1 |  |  |  |  | 1 |  |  |  |  |  |  |  |  |  |  |  |  |  |
| 117 | Nelson et al., 2020 | Responsibility for AI Data Privacy, unnecessary |  |  |  |  |  |  |  |  |  |  |  |  |  |  |  |  |  |  | 1 |  |  |  |  |  |
| 118 | Nelson et al., 2020 | AI Recommendation, Recommended |  |  |  |  |  |  |  |  | 1 |  |  |  |  |  |  |  |  |  |  |  |  |  |  |  |
| 119 | Nelson et al., 2020 | AI Recommendation, Ambivalent |  |  |  |  |  |  |  |  | 1 |  |  |  |  |  |  |  |  |  |  |  |  |  |  |  |
| 120 | Nelson et al., 2020 | AI Recommendation, not recommended |  |  |  |  |  |  |  |  | 1 |  |  |  |  |  |  |  |  |  |  |  |  |  |  |  |
| 121 | Nelson et al., 2020 | AI - Strengths, More objective diagnosis |  |  |  |  |  | 1 |  |  | 1 | 1 | 1 |  |  |  |  |  |  |  |  |  |  |  |  |  |
| 122 | Ngiam and Khor, 2019 | Condition | The long-form text that doctors use to describe a specific condition and its symptoms can be as varied as the doctors themselves. Important considering the context of the clinical history. Conditions, Oncological applications | 1 |  |  |  |  |  |  |  |  |  |  |  |  |  |  |  |  |  |  |  |  |  |  |
| 123 | Ngiam and Khor, 2019 | Data analysis, model building, and validation | 1) Applying appropriate machine learning method for task; 2) Determining cross validation cohorts, 3) Adherence to machine learning best practices as appropriate |  |  | 1 |  |  |  |  |  |  |  |  |  |  |  |  |  |  |  |  |  |  |  | Evaluation of effectiveness |
| 124 | Ngiam and Khor, 2019 | Paper trial | 1) Establishing initial accuracy of alpha model of machine learning tool in prediction task with retrospective test data, 2) Determining effect of tool on human judgment in test setting |  |  |  |  | 1 |  |  |  |  |  |  |  |  |  |  |  |  |  |  |  |  |  |  |
| 125 | Ngiam and Khor, 2019 | Propective clinical trial | 1) Prospective clinical trial of beta model in real world setting with machine learning workflow and clinicians in the loop, 2) Collecting clinical data and assessing effect of machine learning tool in trial setting, 3) Review of clinical trial data and final adjustments of beta model |  |  |  |  | 1 |  |  |  |  |  |  |  |  |  |  |  |  | 1 |  |  |  |  |  |
| 126 | Ngiam and Khor, 2019 | Medical device registration | 1) Design freeze and submission of dossier for medical device registration with appropriate regulatory agencies, 2) Initiate integration with target electronic health record system |  |  |  |  |  |  |  |  |  |  |  |  |  |  |  |  |  | 1 |  |  |  |  |  |
| 127 | Ngiam and Khor, 2019 | Clinical problem (re)definition | 1) Defining clinical problems that are suited to machine learning, 2) Ascertaining mode of human engagement (ie, human-in-the-loop or autonomous), 3) Defining actionable insights |  |  |  |  |  |  | 1 |  |  |  |  |  |  |  |  |  |  |  |  |  |  |  |  |
| 128 | Ngiam and Khor, 2019 | Data extraction selection and refining | 1) Selection of appropriate real world clinical data, 2) Data exploration, 3) Annotation and pre-processing of data |  |  | 1 |  |  |  |  |  |  |  |  |  |  |  |  |  |  |  |  |  |  |  | Types of data inputted |
| 129 | Ngiam and Khor, 2019 | Human-machine interaction | 1) Clinical workflow design involving machine learning tool, 2) Usability of machine learning tool interface, 3) Determining effect of machine learning tool on human decision making, 4) Address legal and ethical implications on practice changes as a result of the machine learning tool |  |  | 1 |  |  |  |  |  | 1 |  |  |  |  |  | 1 |  |  | 1 |  |  |  |  |  |
| 130 | Ngiam and Khor, 2019 | Clinical deployment | 1) Training of clinicians on the use of the machine learning tool and workflow changes before launch, 2) Technical support after launch |  |  |  | 1 |  |  |  |  | 1 |  |  |  |  |  |  | 1 |  |  |  |  |  |  |  |
| 131 | Truong et al., 2019 | Data | AI technology can only be as good as the data used to create it. Thus, factors including data quality, quantity, and collection need to be controlled for. |  |  | 1 |  |  |  |  |  |  |  |  |  |  |  |  |  |  |  |  |  |  |  |  |
| 132 | Truong et al., 2019 | Regulatory strategy | AI technologies that impact patient care will be subject to regulatory oversight in the jurisdiction of commercial use. It is important to have a regulatory strategy during the product development, to ensure a streamlined process. |  |  |  |  |  |  |  |  |  |  |  |  |  |  |  |  |  | 1 |  |  |  |  |  |
| 133 | Truong et al., 2019 | Trust | Trust is generated by understanding, transparency and overall explainability of AI technologies, and is needed by both patients and physicians for effective adoption. |  |  |  |  | 1 |  |  |  |  |  |  |  |  |  |  |  |  |  |  |  |  |  |  |
| 134 | Truong et al., 2019 | Ethics | Ethical challenges arise with the collection and use of patient data, with implementation and dissemination of the technology developed. |  |  |  |  |  |  |  |  | 1 |  |  |  |  |  |  |  |  | 1 |  |  |  |  |  |
| 135 | Truong et al., 2019 | Readiness | The readiness for change of a given clinic or healthcare institution greatly influences the successful implementation of any new practice. Key factors include appropriate infrastucture, sufficient understanding of AI, and effective change management procedures at the institution. |  |  |  |  |  |  |  |  |  |  |  |  | 1 |  |  |  |  |  |  |  |  |  |  |
| 136 | Truong et al., 2019 | Expertise | The input of leaders in the field, including technologists, front-line hospital staff, and clinicians, is required to ensure effective development and implementation of user-friendly technology. |  |  |  |  |  |  |  |  | 1 |  |  |  |  |  |  |  |  |  |  |  |  |  |  |
| 137 | Truong et al., 2019 | Buy-in | Successful implementation is facilitated by generating buy-in from all levels of staff from the very beginning of any given project. All members/stakeholders must understand the value and need of the chosen technology. |  |  |  |  |  |  | 1 |  |  |  |  |  |  |  |  | 1 |  |  |  |  |  |  |  |
| 138 | Truong et al., 2019 | Evaluation | It is essential to have a plan to evaluate the success of the implementation by predefined metrics, including impact on care, medical outcomes or patient experience, integration into workflow, sustainability, and economic considerations. |  |  |  |  | 1 |  |  |  |  |  |  |  |  |  |  | 1 |  |  |  |  |  |  | Evaluation of effectiveness |
| 139 | Truong et al., 2019 | Scalability | Challenges for dissemination and adoption by other clinics or hospitals. ML models trained with local data may have biases that prevent them to be generalizable. |  |  |  |  |  |  |  |  |  |  |  |  |  |  |  |  |  |  |  |  |  | 1 |  |
